# Autologous serum eye drops for patients with dry eye disease: a systematic review and meta-analysis of randomized controlled trials

**DOI:** 10.3389/fmed.2024.1430785

**Published:** 2024-09-13

**Authors:** Chang-Zhu He, Zhao-Jun Zeng, Jun Qiao Liu, Qin Qiu, Yu He

**Affiliations:** ^1^Chengdu University of Traditional Chinese Medicine, Chengdu, China; ^2^Department of Ophthalmology, Chengdu First People's Hospital/Chengdu Integrated TCM and Western Medicine Hospital, Chengdu, China

**Keywords:** autologous serum eye drops, dry eye disease, randomized controlled trials, meta-analysis, systematic review

## Abstract

**Background:**

Dry eye disease (DED) is highly prevalent worldwide, leading to increased medical costs, economic burdens on families and society, and a diminished quality of life for patients. The utilization of autologous serum eye drops (ASEDs) for the treatment of DED is progressively rising.

**Objective:**

To further evaluate the efficacy and safety of ASEDs in the treatment of DED.

**Methods:**

A thorough search for randomized controlled trials (RCTs) was conducted across eight databases, including PubMed, EMBASE, the Cochrane Library, Web of Science, China National Knowledge Infrastructure, Wanfang, SinoMed, and VIP. This search encompassed the inception of each database up to April 1, 2024, with a specific focus on identifying RCTs evaluating the efficacy and safety of ASEDs for the treatment of DED. Data analysis was conducted utilizing Stata 15.0 software and the Cochrane Risk of Bias Assessment Tool was utilized to appraise the literature’s quality.

**Results:**

The study encompassed 12 RCTs. In comparison to the use of artificial tears (AT), patients diagnosed with DED who utilized ASEDs displayed elevated the Schirmer test (ST) scores [WMD = 2.35, 95% CI (1.45, 3.24), *p* < 0.001] and tear-film breakup time (TBUT) scores [WMD = 2.83, 95% CI (2.27, 3.39), *p* < 0.001], decreased Corneal fluorescence staining (CFS) scores [SMD = −2.11, 95% CI (−3.07, −1.15), *p* < 0.001] and the Ocular Surface Disease Index (OSDI) scores [WMD = −10.54, 95% CI (−13.31, −7.77), p < 0.001], and experienced a reduced frequency of adverse events [RR = 0.36, 95% CI (0.13, 0.99), *p* = 0.048].

**Conclusion:**

In this study, ASEDs had been shown to enhance tear secretion, extend tear film break-up time, mitigate corneal epithelial damage, ameliorate OSDI scores, and exhibit greater safety compared to AT.

## 1 Introduction

Dry eye disease (DED) is a multifactorial ocular surface disorder characterized by an imbalance in tear film homeostasis, stemming from either tear deficiency or excessive tear evaporation (1). In DED, dysfunction of the ocular structures responsible for producing and maintaining the tear film components—including the lacrimal glands, meibomian glands, cornea, and conjunctiva—leads to qualitative and/or quantitative tear deficiency, resulting in tear film instability and hyperosmolarity (2). Several factors can influence these structures, potentially contributing to the onset of DED, these factors include ocular diseases like blepharitis and meibomian gland dysfunction, as well as various systemic conditions such as diabetes, Sjögren’s syndrome, rheumatoid arthritis, and systemic lupus erythematosus (3). Dry eye symptoms can be sporadic or persistent, sporadic dry eye can occasionally occur due to environmental conditions and visual tasks that reduce blinking, in contrast, persistent dry eye is characterized by continuous symptoms and ongoing damage to the ocular surface, patients with DED commonly experience symptoms such as grittiness, itching, a sensation of foreign body presence, tearing, burning, visual fatigue, and dryness (4). According to the Tear Film and Ocular Surface Society (TFOS) Dry Eye Workshop II (DEWS II) diagnostic criteria, the global prevalence of dry eye disease is 29.5% (SD = 0.8), with rates of 28.1% (SD = 1.2) in females and 24.9% (SD = 1.4) in males (5). DED significantly impairs patients’ daily activities, including driving, reading, and using electronic screens (computers, televisions, phones) (6). The sleep quality of patients with DED is considerably poorer compared to patients with other inflammatory ocular surface diseases, and this decrease in sleep quality is correlated with the severity of DED (7). Remarkably, in 2011, the direct healthcare costs for managing DED in certain developed countries, such as the United States, were estimated at $3.8 billion annually, with total societal costs reaching approximately $55.4 billion (8). These statistics underscore the significant economic burden that DED places on both individuals and society as a whole.

Currently, artificial tears (AT) are recommended as the first-line treatment for DED, with topical cyclosporine A (CsA) also commonly used for its anti-inflammatory properties, however, these therapies are often inadequate in controlling the signs and symptoms of DED, especially in moderate-to-severe cases (9). Meanwhile, a significant drawback of most AT is their inclusion of preservatives, which have been shown to induce tear film instability, compromise the corneal epithelial barrier, and harm deeper ocular tissues (10). On the contrary, preservative-free eye drops are gentler on the ocular surface, however, they offer relief for only a brief period of 30–40 min and need frequent reapplication (11). A variety of viscosity-enhancing agents are frequently incorporated in AT to enhance lubrication and prolong retention time on the ocular surface, however, high-viscosity eye drops can increase retention time on the ocular surface but may also cause transient visual disturbances and result in unwanted debris on the eyelids and lashes, leading to decreased tolerance and compliance (12). Ocular burning, a frequent side effect of topical CsA with an incidence rate of approximately 17%, frequently leads to patient treatment discontinuation (13).

Autologous serum eye drops (ASEDs) theoretically present a potential advantage over traditional therapies by serving not only as a lacrimal substitute for lubrication but also by containing additional biochemical components that enable them to closely mimic natural tears (14). Similar to tears, ASEDs contain carbohydrates, lipids, various electrolytes, and can also provide vitamins, additionally, ASEDs and tears share a similar osmolarity, approximately 300 mosm/l (15). Several observational studies have found that ASEDs can improve ocular surface dryness and epithelial damage in patients with dry eye syndrome, and they have a high level of safety (16, 17). Currently, several randomized controlled trials (RCTs) have compared the clinical efficacy and safety of ASEDs to AT in treating DED. While some meta-analyses have explored the efficacy of ASEDs in treating DED, this study uniquely examines the frequency of ASEDs usage and the effects of combining ASEDs with AT. Additionally, it conducted a subgroup analysis based on follow-up duration to evaluate the short-term and long-term efficacy of ASEDs. To update the existing data and better evaluate the efficacy and safety of ASEDs to AT in treating DED, we conducted this meta-analysis. Simultaneously, we performed a subgroup analysis based on the frequency of administration, categorizing the subgroups into two categories: six times per day and four times per day, reflecting the most commonly used dosing frequencies. This analysis aimed to determine whether the frequency of administration influences the final outcomes. Furthermore, this study examined the combination of ASEDs with AT and conducted a subgroup analysis to investigate whether the use of ASEDs in conjunction with AT influences the outcomes. Finally, we performed an additional meta-analysis focusing specifically on studies that explicitly identified cases as severe DED, in order to assess the efficacy of ASEDs in treating this severe form of the disease. Our overarching objective is to furnish clinicians and patients with the most appropriate choices in ophthalmic care.

## 2 Methods

This study was registered under PROSPERO (CRD42024539778) adhered to the PRISMA (Preferred Reporting Items for Systematic Reviews and Meta-Analyses) guidelines and was in line with the Cochrane Collaboration’s recommendations (18, 19). Details of the PRISMA checklist can be found in Supplementary material S1.

### 2.1 Search strategy

The two researchers (CZH and ZJZ) independently conducted searches across eight databases, comprising PubMed, EMBASE, the Cochrane Library, Web of Science, China National Knowledge Infrastructure, Wanfang Database, SinoMed, and the VIP Database. The objective was to retrieve eligible literature published from the inception of each database until April 1, 2024. This search did not impose any restrictions based on age, race, region, or language. The search strategy was formulated using Medical Subject Headings (MeSH) along with free terms and a precisely defined set of keywords. For instance, in searching English databases, we chose the following core components: (1) Serum (e.g., Serums, Blood Serum, Serum, Blood); (2) Ophthalmic Solutions (e.g., Solutions, Ophthalmic, Ophthalmic Solution, Solution, Ophthalmic, Eyedrops, Eye Drops, Drops, Eye, Eye Drop, Drop, Eye, Eyedrop); (3) Dry Eye Syndromes (e.g., Dry Eye Syndrome, Dry Eye Disease, Dry Eye Diseases, Dry Eye, Dry Eyes, Evaporative, Dry Eye Disease, Evaporative Dry Eye Syndrome, Evaporative Dry Eye, Dry Eye, Evaporative, Evaporative Dry Eyes). Additionally, relevant articles from initial search meta-analyses and gray literature were reviewed and included. The search for gray literature was conducted by two researchers (CZH and ZJZ), primarily focusing on accessible master’s and doctoral theses from Chinese universities available in CNKI. Detailed retrieval procedures are outlined in Supplementary material S2.

### 2.2 Inclusion criteria

1. Type of study: This meta-analysis included only RCTs of ASEDs in the treatment of DED.
2. Type of participants: Patients diagnosed with DED should adhere to authoritative clinical practice guidelines, such as TFOS DEWS II Diagnostic Methodology report or Concordance between Chinese dry eye diagnostic criteria and Asian dry eye diagnostic criteria (20, 21).
3. Type of interventions and controls: In all included studies, patients with DED in the experimental group received treatment with ASEDs (either alone or in combination with AT), while patients in the control group received AT only.
4. Type of outcomes: Included studies examined at least one of the following outcomes:
  - The Schirmer test (ST): ST assesses tear volume in patients with DED by inserting strips into the lower tear meniscus. Elevated ST scores indicate less severe symptoms of DED, whereas lower scores suggest more pronounced symptoms (22).
  - Tear-film breakup time (TBUT): TBUT evaluates tear film stability by measuring the time until tear film rupture following local instillation of sodium fluorescein. A shorter TBUT suggests decreased tear film stability (23).
  - Corneal fluorescence staining (CFS): The CFS score entails dividing the cornea into four quadrants and summing the scores from each quadrant, resulting in a total score ranging from 0 to 12 points (24). However, despite its widespread use, a universally accepted “gold standard” grading scale for corneal and conjunctival staining does not exist (25). For example, Begley et al. (25) developed a corneal fluorescein staining scale that divides the cornea into five zones: central, superior, inferior, nasal, and temporal. Miyata et al.’s (26) corneal grading scale divides corneal staining into two attributes: area and density. Considering the use of different CFS scales in the studies included in this research, we analyzed the data using the standard mean difference (SMD).
  - The Ocular Surface Disease Index (OSDI): OSDI was used to examine general ocular-related symptoms for patients with dry eye disease. The total OSDI score ranged between 0 and 100 points. A higher OSDI score means more serious symptoms of dry eye disease (27). According to a report from TFOS DEWS II, the OSDI is the most widely used questionnaire for DED clinical trials, it measures the frequency of symptoms, environmental triggers, and vision-related quality of life. Many other questionnaires have recently established concurrent validity against the OSDI (20).
  - Adverse events: Adverse events include post-medication ocular pain, itching, and foreign body sensation, as well as conjunctival congestion and swelling.
5. Types of severe DED: For the analysis of severe DED, we conducted a separate study. To ensure the accuracy of our findings, specific inclusion criteria were established for participants with severe DED. The definition of severe DED required meeting at least one of the following criteria: an OSDI score exceeding or equal to 33, a TBUT less than 5 s, or a ST score below 5 (20).

### 2.3 Exclusion criteria

Exclusion criteria included the following aspects: (1) Any non-RCTs were excluded, including animal experiments, conference reports, reviews, retrospective studies, meta-analyses, case reports, etc. (2) Patients diagnosed with DED were treated with various eye drops apart from ASEDs and AT, encompassing steroid eye drops, anti-inflammatory eye drops or antibiotic eye drops. (3) RCTs without relevant outcomes or complete data were not available.

### 2.4 Data extraction

The two authors (CZH and ZJZ) initially screened the titles and abstracts of the literature retrieved through preliminary searches according to specified inclusion and exclusion criteria. Subsequently, the remaining literature was thoroughly reviewed based on predefined inclusion and exclusion criteria, with studies meeting the inclusion criteria being ultimately selected. For the eligible studies, the two authors (CZH and ZJZ) independently extracted the following information: author, year, region, sample size, patient age, detailed intervention and control measures, outcome indicators, and treatment duration. In cases where consensus could not be reached through discussion, the third author (YH) was consulted. If data were missing from the included literature, efforts were made to contact either the first author or the corresponding author to obtain the necessary information.

### 2.5 Assessment of risk of bias

The risk of bias in the included studies was independently assessed by two authors (CZH and SJL) using the Cochrane Risk of Bias Version 2 (RoB 2) assessment tool (28). RoB 2 evaluates six key aspects: (1) random sequence generation, (2) deviations from intended interventions, (3) missing outcome data, (4) outcome measurement, (5) selection of reported results, and (6) overall bias. Each aspect is categorized as “low risk,” “some concern,” or “high risk,” depending on the specific circumstances of the study.

### 2.6 Statistical analysis

Stata 15.0 software was used to conduct the meta-analysis. SMD or weighted mean difference (WMD) with 95% confidence intervals were employed to analyze continuous variables. For binary variables, the risk ratio (RR) and its corresponding 95% confidence interval were utilized for analysis. Given the potentially significant differences in research methods across studies and the varying baseline conditions of patients with DED, as well as the diversity in methods used to prepare ASEDs, there is considerable clinical heterogeneity evident in the studies included. Therefore, regardless of the statistical heterogeneity, we used a random-effects model to analyze the data. Simultaneously, to further explore the therapeutic effects of ASEDs, subgroup analyses were conducted based on the frequency of ASEDs use and whether they were combined with AT. Given the wide range of follow-up durations in the included studies, we conducted a subgroup analysis by dividing the follow-up periods into multiple stages. The aim was to investigate whether the length of the follow-up period affects the outcomes and to assess the effectiveness of ASEDs in treating DED at different time points.

### 2.7 Sensitivity analysis

To evaluate the robustness of the results, a sensitivity analysis was conducted whereby studies were sequentially excluded. If an exclusion resulted in a significant influence on the outcome and a reversal of the conclusion, the full text of the article was thoroughly analyzed to determine if it was a source of heterogeneity. Conversely, in the absence of a reversal, the results were deemed robust.

### 2.8 Assessment of publication bias

When the number of included studies reached 10 or more, a funnel plot was utilized, and visual inspection was employed to assess potential publication bias. The Begg’s test and the Egger’s test were then employed to further examine publication bias.

## 3 Results

### 3.1 Study selection

**Figure 1:**
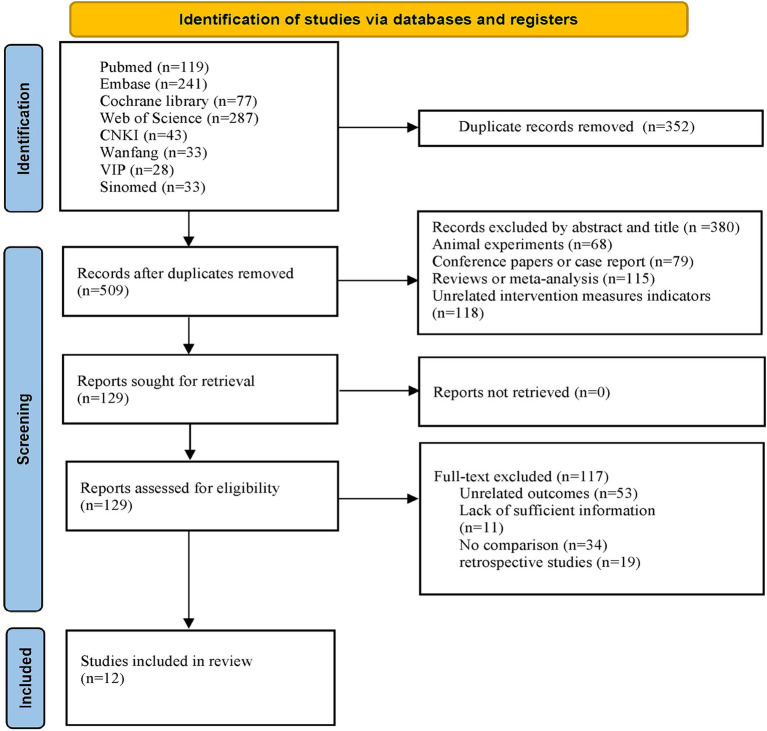
Flow chart of selection studies and specific reasons for exclusion.

Through the initial search, a total of 861 articles were retrieved from eight databases. Following the removal of duplicates using EndNote software, 509 articles remained. After reviewing the titles and abstracts of the articles, an additional 380 papers were excluded. Among the excluded literature were 68 animal experiments, 79 conference reports or case reports, 115 reviews or meta-analyses, and 118 papers with inappropriate interventions. After thoroughly reviewing the full texts of the remaining 129 articles, 53 were deemed ineligible due to insufficient relevant outcome indicators, 11 were excluded owing to incomplete data, 34 were dismissed for lacking control groups, and 19 retrospective studies were excluded. Ultimately, 12 articles meeting the eligibility criteria were retained for analysis. The detailed procedure for literature screening is delineated in Figure 1.

### 3.2 Study characteristics

In the selection of the 12 included studies (29–40), all were identified as RCTs. Six studies (35–40) originated from China, followed by two from Turkey (30, 32), one from Japan (29), one from Chile (31), one from Italy (34), and one from India (33). The sample sizes included in the literature range from 10 to 144, spanning from 2005 to 2024. In terms of intervention measures, all control groups included in the literature used AT (such as sodium hyaluronate eye drops and polyethylene glycol eye drops), while the experimental groups in four studies used ASEDs combined with AT, with the remaining studies using ASEDs alone. In the context of usage frequency, four studies employed a dosing regimen of four times per day, seven studies utilized a regimen of six times per day, while one study did not provide specific details. One study (32) used ASEDs at a concentration of 40%, one study (34) did not report the concentration of the eye drops, while the remaining studies all used ASEDs at a concentration of 20%. In terms of treatment duration, the longest course lasted for 12 months, while the shortest course was 2 weeks. A total of four articles (30, 31, 33, 35) explicitly mentioned targeting patients with severe DED, the remaining articles did not explicitly mention the severity of DED patients or did not specify the criteria used to define severe DED. The characteristics of the included studies were detailed in Table 1.

**Table 1:** Basic characteristics of the included studies.

References	Region	Year	Sample(T/C)	Age		Interventions		Frequency of ASEDs	Concentration	Duration	Outcome
				T	C	T	C				
Kojima T, et al.	Japan	2005	10/10	65.4 ± 9.7	62.3 ± 12.5	ASEDs	AT	6 times/d	20%	2 weeks	TBUT, CFS, OSDI
Celebi AR, et al.	Turkey	2014	20/20	56.05 ± 8.07		ASEDs	AT	4 times/d	20%	6 weeks	OSDI
Urzua CA, et al.	Chile	2012	12/12	52 ± 6.3		ASEDs	AT	4 times/d	20%	5 weeks	TBUT, OSDI
Yılmaz U, et al.	Turkey	2017	24/24	25 ± 4.02		ASEDs	AT	4 times/d	40%	2 months	ST, TBUT
Mukhopadhyay S, et al.	India	2015	52/44	NR		ASEDs	AT	6 times/d	20%	6 weeks	ST, TBUT, CFS
Semeraro F, et al.	Italy	2016	12/12	54.6 ± 15.4	54.0 ± 7.2	ASEDs	AT	NR	NR	12 months	TBUT, OSDI
Zheng N, et al.	China	2023	116/116	54.6 ± 12.4	55.2 ± 11.8	ASEDs	AT	4 times/d	20%	12 weeks	ST, TBUT, CFS, OSDI
Lu ZM, et al.	China	2023	39/39	69.1 ± 4.8	65.1 ± 10.5	ASEDs+AT	AT	6 times/d	20%	4 weeks	ST, TBUT, CFS
Yao T, et al.	China	2020	21/21	NR		ASEDs	AT	6 times/d	20%	4 weeks	ST, TBUT, CFS, OSDI
Kang HJ, et al.	China	2021	48/48	62.0 ± 6.6	64.0 ± 6.6	ASEDs+AT	AT	6 times/d	20%	1 month	ST, TBUT, CFS, Adverse events
Ma WT, et al.	China	2022	62/62	61.8 ± 10.2	62.4 ± 9.8	ASEDs+AT	AT	6 times/d	20%	4 weeks	ST, TBUT
Zhou AI, et al.	China	2024	144/144	70.9 ± 5.9	71.3 ± 6.0	ASEDs+AT	AT	6 times/d	20%	1 month	ST, TBUT, OSDI, Adverse events

NR, not report; T, test group; C, control group; ST, The Schirmer test; TBUT, Tear-film breakup time; CFS, Corneal fluorescence staining; OSDI, The Ocular Surface Disease Index; ASEDs, Autologous serum eye drops; AT, Artificial tears.

### 3.3 Risk of bias

**Figure 2:**
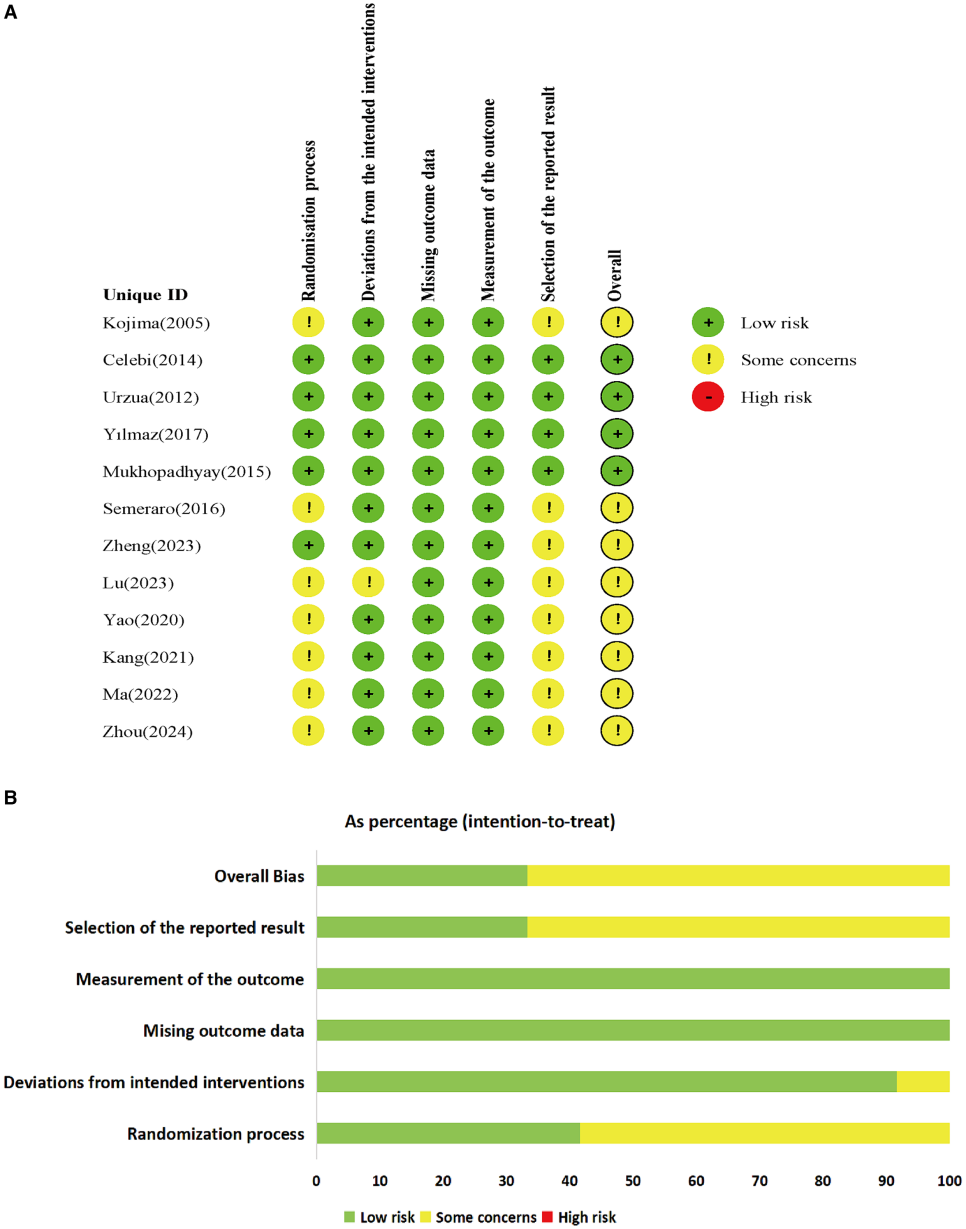
Risk of bias of RCTs; **(A)** Risk of bias graph; **(B)** Risk of bias summary.

Among the 12 included studies, none were deemed to pose a high risk, while 4 studies (30–33) were categorized as low risk. Two studies (33, 35) employed computer-generated sequences for random allocation, whereas four studies (36–38, 40) utilized random number tables for allocation, the remaining studies mentioned random allocation without providing specific details regarding the allocation method. Among the five studies (30–33, 35), double-blinding was explicitly mentioned, whereas the remaining studies lacked specific explanations regarding the blinding method utilized. Among the included studies, one study (35) had missing data, while the data in the rest of the studies were complete. The missing data in that particular study fell within an acceptable range and were determined to have no substantial effect on the final outcomes. None of the articles deviated from the expected interventions, nor did they selectively report results. The risk of bias in the included RCTs is listed in Figure 2.

### 3.4 Meta-analysis results

#### 3.4.1 St

**Figure 3:**
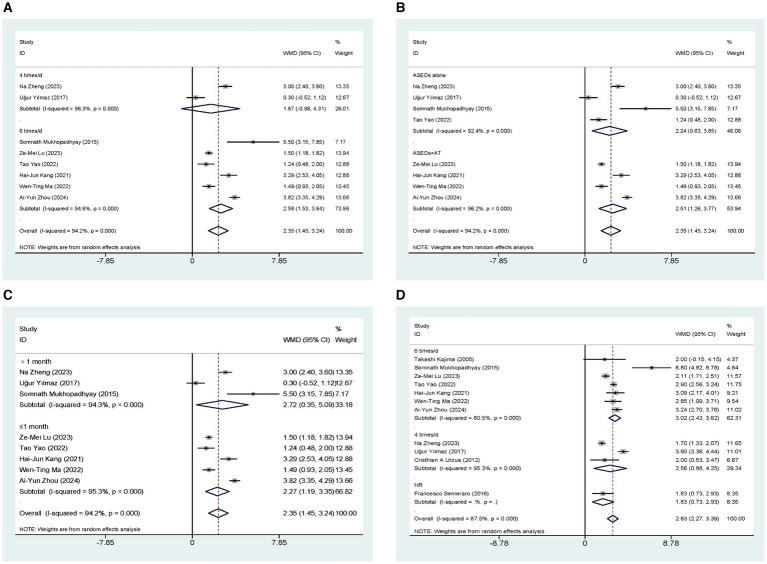
Forest plot of the comparison results; **(A)** The ST results of subgroups were analyzed based on the frequency of ASEDs usage; **(B)** The ST results of subgroups were analyzed based on whether ASEDs were combined with AT; **(C)** The ST results of subgroups were analyzed based on follow-up period; **(D)** The TBUT results of subgroups were analyzed based on the frequency of ASEDs usage.

Eight studies (32, 33, 35–40) conducted a comparative analysis on the ST scores of patients administered with ASEDs and AT. The resultant summary statistics demonstrated that ASEDs yielded significantly higher ST scores than AT [WMD = 2.35, 95% CI (1.45, 3.24), *p* < 0.001] (Figure 3A). Through subgroup analysis based on frequency of use, it was discovered that patients using ASEDs six times daily exhibited significantly higher ST scores compared to those using AT [WMD = 2.58, 95% CI (1.53, 3.64), *p* < 0.001], whereas there was no statistical significance in the ST scores of patients using ASEDs four times daily compared to those using AT [WMD = 1.67, 95% CI (−0.98, 4.31), *p* = 0.217] (Figure 3A). Based on whether ASEDs were used in combination with AT, subgroup analysis revealed that both the combined group and the single drug group exhibited significantly higher ST scores compared to AT ([WMD = 2.51, 95% CI (1.26, 3.77), *p* < 0.001] and [WMD = 2.24, 95% CI (0.63, 3.85), *p* = 0.006] respectively) (Figure 3B). We also conducted a subgroup analysis based on whether the follow-up period exceeded 1 month. The results indicated that both subgroups, with follow-up periods of less than or equal to 1 month and more than 1 month, showed significant differences compared to the AT group ([WMD = 2.27, 95% CI (1.19, 3.35), *p* < 0.001] and [WMD = 2.72, 95% CI (0.35, 5.09), *p* = 0.025] respectively) (Figure 3C).

#### 3.4.2 TBUT

**Figure 4:**
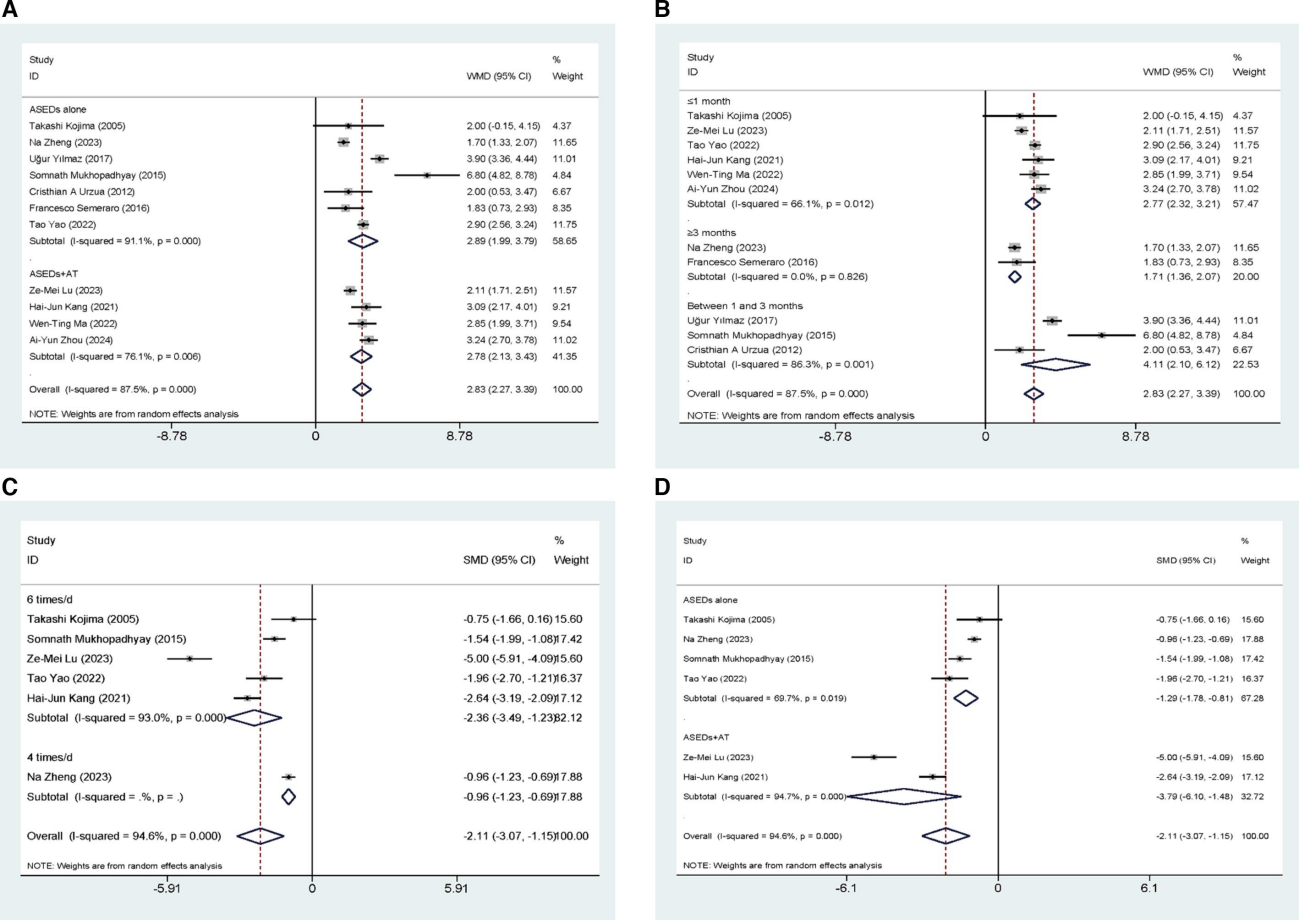
Forest plot of the comparison results; **(A)** The TBUT results of subgroups were analyzed based on whether ASEDs were combined with AT; **(B)** The TBUT results of subgroups were analyzed based on follow-up period; **(C)** The CFS results of subgroups were analyzed based on the frequency of ASEDs usage; **(D)** The CFS results of subgroups were analyzed based on whether ASEDs were combined with AT.

Eleven studies (29, 31–40) conducted comparisons of TBUT scores between ASEDs and AT. The summary results indicated that ASEDs yielded significantly higher TBUT scores compared to AT [WMD = 2.83, 95% CI (2.27, 3.39), *p* < 0.001] (Figure 3D). Through subgroup analysis based on frequency of use, it was discovered that both the use of ASEDs six times a day and the use of ASEDs four times a day resulted in significantly higher TBUT scores compared to AT ([WMD = 3.02, 95% CI (2.43, 3.62), *p* < 0.001] and [WMD = 2.56, 95% CI (0.88, 4.25), *p* = 0.003] respectively) (Figure 3D). Based on whether ASEDs were used in combination with AT, subgroup analysis revealed that both the combined group and the single drug group exhibited significantly higher TBUT scores compared to AT ([WMD = 2.78, 95% CI (2.13, 3.43), *p* < 0.001] and [WMD = 2.89, 95% CI (1.99, 3.79), *p* < 0.001] respectively) (Figure 4A). A subgroup analysis based on follow-up periods revealed that the ASEDs group exhibited significant differences compared to the AT group when the follow-up period was less than or equal to 1 month, between one and 3 months, and greater than 3 months ([WMD = 2.77, 95% CI (2.32, 3.21), *p* < 0.001], [WMD = 4.11, 95% CI (2.10, 6.12), *p* < 0.001] and [WMD = 1.71, 95% CI (1.36, 2.07), *p* < 0.001] respectively) (Figure 4B).

#### 3.4.3 CFS

**Figure 5:**
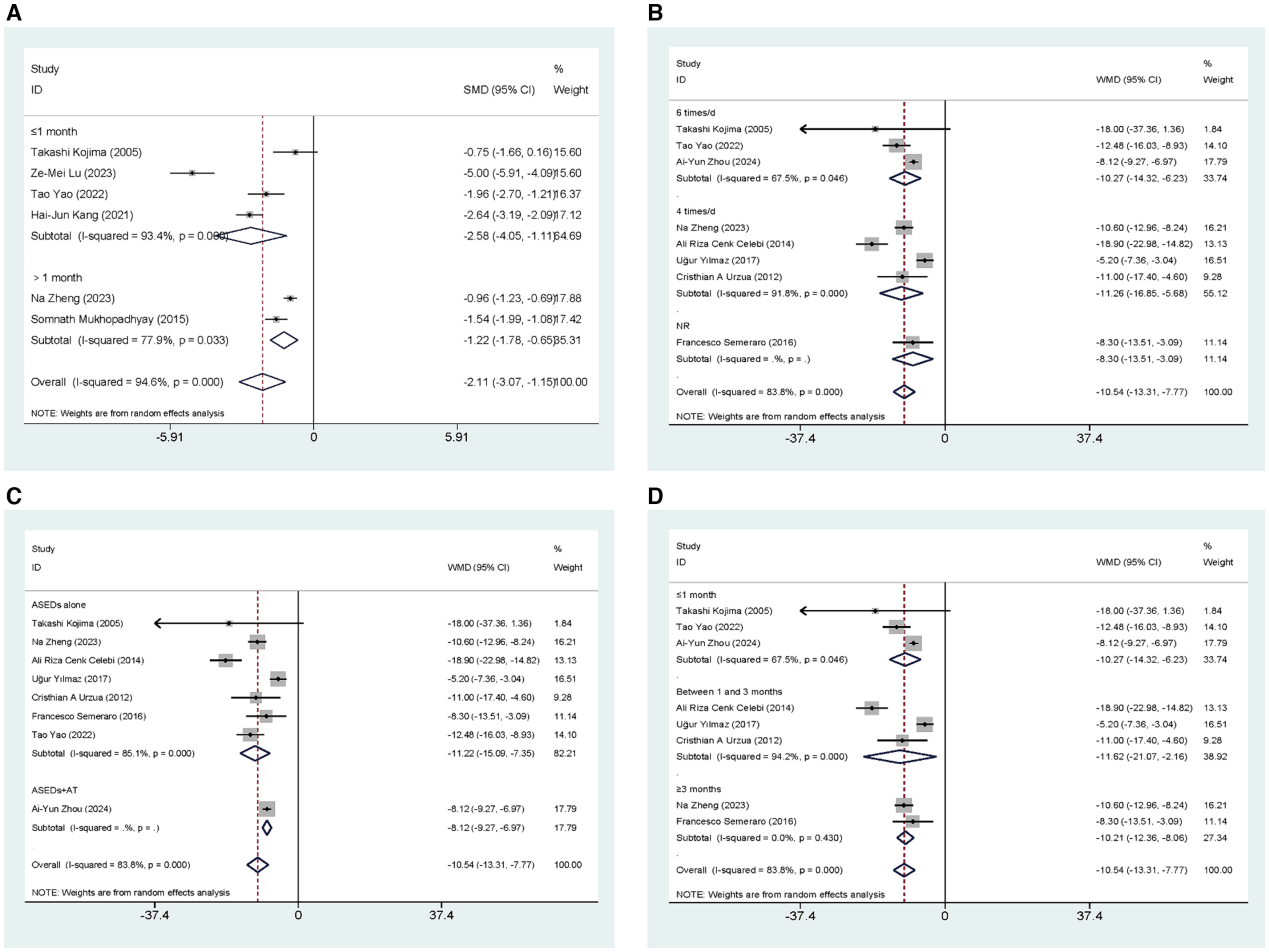
Forest plot of the comparison results; **(A)** The CFS results of subgroups were analyzed based on follow-up period; **(B)** The OSDI results of subgroups were analyzed based on the frequency of ASEDs usage; **(C)** The OSDI results of subgroups were analyzed based on whether ASEDs were combined with AT; **(D)** The OSDI results of subgroups were analyzed based on follow-up period.

Six studies (29, 33, 35–38) conducted comparisons of CSF scores between ASEDs and AT. The aggregated findings indicated that ASEDs exhibited significantly lower CSF scores compared to AT [SMD = −2.11, 95% CI (−3.07, −1.15), *p* < 0.001] (Figure 4C). Based on whether ASEDs were used in combination with AT, subgroup analysis revealed that both the combined group and the single drug group exhibited significantly lower CFS scores compared to AT ([SMD = −3.79, 95% CI (−6.10, −1.48), *p* = 0.001] and [SMD = −1.29, 95% CI (−1.78, −0.81), *p* < 0.001] respectively) (Figure 4D). A subgroup analysis based on follow-up periods showed that the ASEDs group exhibited significant differences compared to the AT group when the follow-up period was less than or equal to 1 month and more than 1 month ([SMD = −2.58, 95% CI (−4.05, −1.11), p = 0.001] and [SMD = −1.22, 95% CI (−1.78, −0.65), *p* < 0.001] respectively) (Figure 5A).

#### 3.4.4 OSDI

Eight studies (29–32, 34, 35, 37, 40) conducted comparisons of OSDI scores between ASEDs and AT. The summary results showed that compared to AT, ASEDs had significantly lower OSDI scores [WMD = −10.54, 95% CI (−13.31, −7.77), *p* < 0.001] (Figure 5C). Subgroup analysis based on usage frequency revealed that the OSDI scores were significantly lower with the use of ASEDs six times daily and ASEDs four times daily, compared to AT ([WMD = −10.27, 95% CI (−14.32, −6.23), *p* < 0.001] and [WMD = −11.26, 95% CI (−16.85, −5.68), *p* < 0.001] respectively) (Figure 5B). A subgroup analysis based on follow-up periods revealed that the ASEDs group exhibited significant differences compared to the AT group when the follow-up period was less than or equal to 1 month, between one and 3 months, and greater than 3 months ([WMD = −10.27, 95% CI (−14.32, −6.23), *p* < 0.001], [WMD = −11.62, 95% CI (21.07, −2.16), *p* = 0.016] and [WMD = −10.21, 95% CI (−12.36, −8.06), *p* < 0.001] respectively) (Figure 5D).

#### 3.4.5 Adverse events

**Figure 6:**
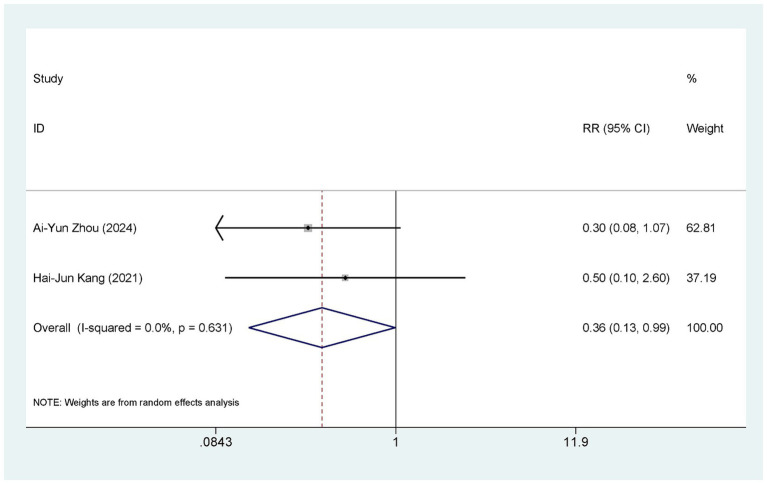
Forest plot of the comparison results of adverse events.

Two studies (38, 40) compared the occurrence of adverse events between ASEDs and AT. Two studies (38, 40) reported the incidence of conjunctival hyperemia, two studies (38, 40) reported the incidence of itchy eyes, two studies (38, 40) reported the incidence of eye swelling, and one study (38) reported the incidence of corneal infection. Detailed information is provided in Table 2. In summary, the ASEDs group reported an adverse reaction rate of 2.60% (5/192), whereas the AT group reported an adverse reaction rate of 7.80% (15/192). The pooled results showed that the incidence of adverse events with ASEDs was significantly lower than with AT [RR = 0.36, 95% CI (0.13, 0.99), *p* = 0.048] (Figure 6).

**Table 2:** The incidence rate of adverse events.

Adverse effect	References	Total number of adverse effects	
		Test group	Control group
Conjunctival hyperemia	Kang et al. (38), Zhou et al. (40)	1	5
Eye itch	Kang et al. (38), Zhou et al. (40)	2	4
Eyes red and swollen	Kang et al. (38), Zhou et al. (40)	2	4
Corneal infections	Kang et al. (38)	0	1
Total events		5/192	15/192
Incident rate		2.60%	7.80%

#### 3.4.6 Outcomes related to severe DED

**Figure 7:**
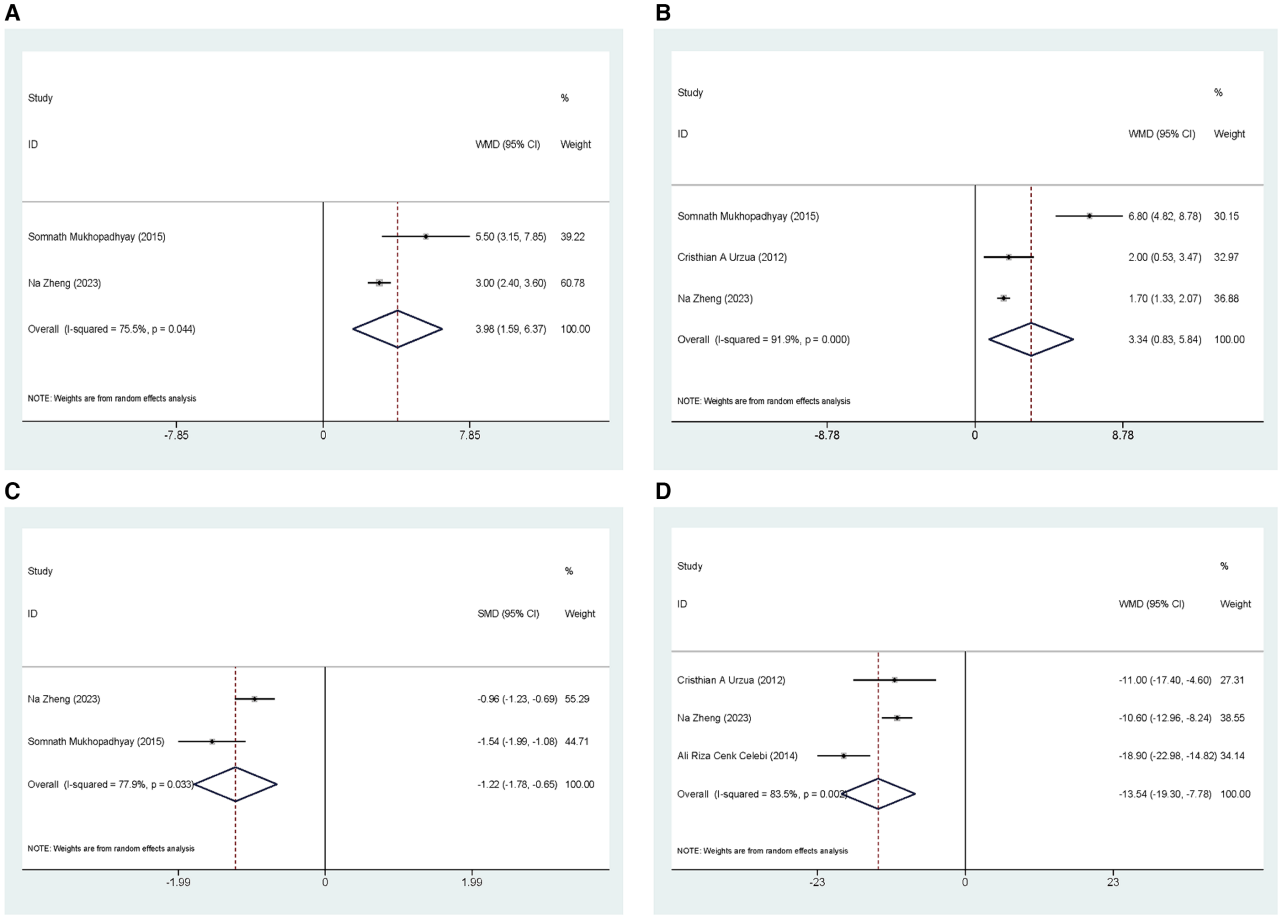
Outcomes related to severe DED: **(A)** Forest plot results of the ST; **(B)** Forest plot results of the TBUT; **(C)** Forest plot results of the CFS; **(D)** Forest plot results of the OSDI.

Two studies compared the effects of ASEDs on ST scores in the treatment of severe DED. The pooled results indicated that ASEDs improve ST scores in DED patients compared to AT [WMD =3.98, 95% CI (1.59, 6.37), *p* = 0.001] (Figure 7A). Three studies compared TBUT scores. The pooled results indicated that ASEDs can improve TBUT scores in patients with severe DED compared to AT [WMD =3.34, 95% CI (0.83, 5.84), *p* = 0.009] (Figure 7B). Two studies compared CFS scores. The pooled results indicated that ASEDs can lower CFS scores in patients with severe DED compared to AT [SMD = -1.22, 95% CI (−1.78, −0.65), *p* < 0.001] (Figure 7C). Three studies compared OSDI scores. The pooled results indicated that, compared to AT, ASEDs can reduce OSDI scores in patients with severe DED [WMD = −13.54, 95% CI (−19.30, −7.78), *p* < 0.001] (Figure 7D).

### 3.5 Sensitivity analysis

**Figure 8:**
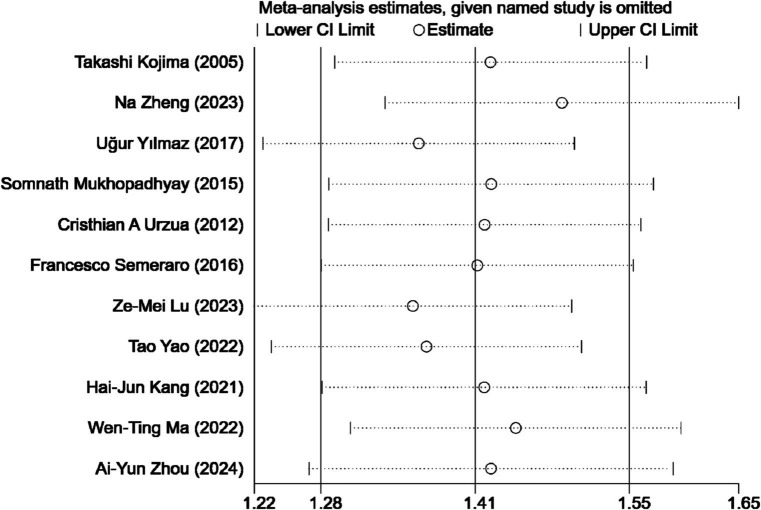
Sensitivity analysis.

A sensitivity analysis was conducted to assess the influence of individual studies on the overall outcome of our study comparing ASEDs with AT for DED. The findings revealed that no single study significantly impacted the final results, suggesting the robustness and stability of the study’s findings (Figure 8).

### 3.6 Publication bias

For outcomes with more than 10 included studies, we visually inspected funnel plots to explore the potential for publication bias, the funnel plots appeared slightly asymmetrical. To go further with the exploration, we used Begg’s test and Egger’s test. Begg’s test yielded a *p* value of 0.755, and Egger’s test yielded a *p* value of 0.401, suggesting that publication bias is unlikely to have significantly influenced the results of this study.

## 4 Discussion

The present meta-analysis aimed to explore the effectiveness and safety of ASEDs as a treatment for DED. The final results indicated that ASEDs demonstrated a remarkable improvement in both ST and TBUT scores among DED patients, while simultaneously decreasing scores on the OSDI and the CFS. Simultaneously, based on the analysis of safety considerations, we discovered a lower occurrence of adverse events associated with ASEDs, which suggested a comparatively superior safety profile. In order to further investigate the optimal usage of ASEDs, subgroup analysis was conducted. Typically, clinicians recommend using ASEDs four times daily or six times daily. Consequently, we conducted subgroup analyses to explore the effects of varying usage frequencies. The subgroup analysis revealed that ASEDs administered six times daily showed significant enhancement in ST scores, while ASEDs administered four times daily seemed to be ineffective in improving ST scores. However, both groups demonstrated effectiveness in improving TBUT scores and reducing OSDI scores. At the same time, we conducted subgroup analysis based on whether ASEDs were used in conjunction with AT. The results revealed that both ASEDs combined with AT and ASEDs used alone demonstrated improvements in ST and TBUT scores, concurrently reducing CFS scores. Meanwhile, due to the wide range of follow-up durations in the included studies, we conducted a subgroup analysis based on follow-up periods to ensure the stability of the results and to explore the short-term and long-term efficacy of ASEDs in treating DED. The final results indicated that ASEDs can effectively improve the symptoms of DED patients both in the short term and the long term, compared to AT.

The meta-analysis results by Wang et al. (41) indicated that ASEDs can enhance TBUT and OSDI scores in DED patients, which is consistent with our study findings. A network meta-analysis conducted by Jongkhajornpong et al. (42) investigated the efficacy of various biological tear substitutes in treating DED. The analysis revealed that umbilical cord serum is the most effective treatment for extending TBUT, whereas autologous platelet lysate demonstrates the greatest efficacy in reducing the OSDI. Through subgroup analysis of follow-up time, we found results similar to those of a meta-analysis by Franchini et al. (43), indicating that ASEDs have a significant effect on improving TBUT and OSDI outcomes in the short term (within 6 weeks). However, while the meta-analysis by Quan et al. (44). supported the efficacy of ASEDs in improving symptoms after 2 weeks of treatment, there was no evidence to indicate that they result in symptom improvement in DED patients after 4 weeks. In a retrospective study by Hussain et al. (45), it was observed that ASEDs can enhance ST and OSDI scores in patients with DED during a mean follow-up period of 1 year. For severe DED, we conducted a separate meta-analysis of the outcomes. The pooled results showed that ASEDs have a significant improvement effect on severe DED. In a study by Rybickova et al. (46), it was also found that a three-month course of autologous serum treatment led to the improvement of ocular surface apoptosis, particularly in patients with severe DED attributed to graft versus host disease. In the subgroup analysis, both the utilization of ASEDs independently and their combination with AT demonstrated positive outcomes. Inflammation plays a key role in the pathophysiology of DED, DED-related inflammation involves both innate and adaptive immune responses. The innate response begins when environmental stress disrupts the tear film and associated ocular structures, triggering a signaling cascade mediated by mitogen-activated protein kinases (MAPK) in ocular surface epithelial cells. This activates transcription factors like nuclear factor kappa B (NF-κB), activator protein 1 (AP-1), and activating transcription factor (ATF), leading to the release of pro-inflammatory cytokines, chemokines, and matrix metalloproteinases (MMPs). The inflammatory environment then activates immature antigen-presenting cells (APCs), which mature and migrate to regional lymphoid tissues, initiating the adaptive immune response (47). Activating inflammatory pathways and releasing inflammatory factors lead to a vicious DED cycle, therefore, anti-inflammatory therapy seems to be a more appropriate approach for treating dry eye disease (48). ASEDs contain a series of cytokines, such as interleukin-1 receptor antagonist, tumor necrosis factor-alpha, interferon-gamma, and interleukin-6 (49). In addition, ASEDs contain essential vitamins such as vitamin A and E; potent antioxidants such as glutathione; as well as numerous other bioactive molecules including fibronectin, albumin, immunoglobulins, and lysozyme, which can regulate ocular surface inflammation and immune responses, inhibit pro-inflammatory mediators, and reduce oxidative stress (50). In DED, the absence of growth factors, vitamins, and neuropeptides in tears hampers the proliferation, migration, and differentiation of ocular surface epithelium, leading to persistent epithelial defects. In this situation, lubricating the ocular surface is crucial, however, the ideal tear substitute should also offer epitheliotrophic support (51). The growth and migration-promoting effects of serum on cell cultures, including corneal epithelial cells, have been extensively documented (52). Human serum contains several substances that play critical roles in tissue repair processes. Among these are epithelial growth factor (EGF), which accelerates epithelial cell migration and exhibits antiapoptotic effects, and transforming growth factor β (TGF-β), which is integral to both epithelial and stromal repair mechanisms (53). In addition, autologous serum also contains neuronal factors, including substance P (SP) and insulin-like growth factor 1 (IGF-1), which appear to play a role in the migration and adhesion of corneal epithelial cells (54). Tsubota discovered that serum supports the migration of an SV40-transfected human corneal epithelial cell line in a dose-dependent manner, additionally, immortalized conjunctival epithelial cells, indicative of higher differentiation, begin to express mucin-1 (50). Lekhanont et al. (55), found in a study involving 181 patients with postoperative corneal epithelial defects that the overall success rate of ASEDs in treating persistent postoperative epithelial defects was 93.92% (95% CI 0.88–0.98). Meanwhile, ASEDs contain immunoglobulins (such as IgG and IgA) and lysozymes, which exhibit both bactericidal and bacteriostatic effects (56).

Our study revealed that the utilization of ASEDs six times per day exhibits greater efficacy in enhancing ST scores compared to their administration four times a day, which may be related to the concentration of the prepared ASEDs. Currently, there is no consensus on the standard protocol for the preparation of autologous serum eye drops. Various studies have reported the utilization of concentrations ranging from 20 to 100% (57). In our included studies, only one article used ASEDs with a concentration of 40%, while the rest used 20%. The most commonly utilized concentration in previous studies is 20%. Serum is typically diluted 1:5 to reduce the concentration of TGF-β to a level comparable to that found in natural tears. This is because the initial study, which used low centrifugation speed, found a fivefold higher concentration of TGF-β in serum compared to tears (55). However, the subsequent study employed a higher centrifugation force for serum preparation, leading to a significantly lower concentration of TGF-β compared to the findings of the earlier report (51). Kumari et al. (58) compared 20 and 50% ASEDs in a randomized controlled trial, and the results revealed that for severe DED, the 50% ASEDs had a more favorable effect in improving subjective symptoms. Higher-concentration ASEDs contain a higher abundance of SP, insulin-like growth factors, cytokines, and vitamins, potentially offering a more favorable environment for repairing the ocular surface microenvironment. In a study conducted by Cho et al. (59), it was found that in eyes affected by Sjögren’s syndrome with persistent epithelial defects, 100% ASEDs was the most effective treatment in reducing symptoms, improving corneal epitheliopathy, and promoting rapid wound closure. Wróbel-Dudzińska et al. (60) also investigated the effects of 100% ASEDs on treating DED in patients with primary Sjögren’s syndrome, the study reported statistically significant improvements in best-corrected visual acuity (BCVA), ST scores, TBUT, and meibomian gland parameters, as well as reductions in OSDI scores, Oxford staining, and conjunctival hyperemia. While evidence from cell cultures suggests that diluting serum to 20% or less enhances epithelial cell proliferation, epithelial migration and extracellular matrix deposition by fibroblasts are better stimulated by serum concentrations of 50% or 100% (12). Regarding safety, the occurrence of adverse events in the ASEDs group was comparatively lower, which could be attributed to the similarity between ASEDs and unstimulated human tears in terms of pH value (7.4) and osmolarity (296–8 mOsm/kg H_2_O) (51). In addition, the presence of preservatives in artificial tears has the potential to trigger adverse events.

The current study revealed that the majority of the included literature originated from Asia, primarily focusing on China. According to a 2017 report by TFOS DEWS II, individuals of Asian ethnicity are considered a significant risk factor for DED (61). A study by Song et al. (62) indicated that in China, an estimated 170.09 million people suffer from DED due to symptoms and signs, while 394.13 million people suffer from symptomatic DED alone. This might elucidate why a significant portion of cases originated from Asia, particularly China.

The meta-analysis possesses several notable advantages: Firstly, it exclusively incorporated RCTs, guaranteeing consistency in the study types. Secondly, this study performed a subgroup analysis based on the frequency of administration and found that administering ASEDs six times per day resulted in a more significant improvement in ST scores compared to four times per day. Additionally, the analysis on the combination of ASEDs with AT demonstrated that both the standalone use of ASEDs and their combination with AT produced favorable outcomes. Thirdly, a separate meta-analysis was performed on studies specifically identifying cases as severe DED, with the final results demonstrating that ASEDs are effective in treating this severe form of the condition. This study also presents certain limitations. To begin with, our study included only Chinese and English literature, and the majority of studies included in this research were from a single region, thus casting doubt on the global applicability of ASEDs. Secondly, due to the fact that almost all of the studies included in this research utilized 20% ASEDs, it was not possible to carry out subgroup analysis or discussion pertaining to the concentration of ASEDs. Thirdly, in the analysis of related adverse event outcomes, only two studies were included. Due to the limited number of included studies, this outcome may not fully reflect the true incidence of related adverse events, and therefore, the results should be interpreted with caution.

## 5 Conclusion

Overall, ASEDs demonstrated impressive effectiveness when used alone or in combination with AT to improve ST and TBUT scores in individuals with DED. Furthermore, they significantly decreased OSDI and CFS scores while maintaining a high level of safety. Moreover, the efficacy of using ASEDs six times per day appeared to surpass that of using them four times daily, particularly in terms of significantly improving ST scores. For severe DED, ASEDs also demonstrated superior efficacy compared to AT. Therefore, the use of ASEDs presents a viable option for addressing DED due to their favorable efficacy and safety profiles.

## Data Availability

The original contributions presented in the study are included in the article/Supplementary material, further inquiries can be directed to the corresponding author.
